# Serotonin 5-HT7 receptor signaling modulates inflammatory responses and survival after myocardial infarction

**DOI:** 10.1186/s12967-026-08423-4

**Published:** 2026-06-17

**Authors:** Franziska E. Müller, Frauke S. Bahr, Sergej Erschow, Martina Kasten, Maren Heimerl, Nils Benen, Mira Jung, Arne Schmidt, Michaela Scherr, Christine S. Falk, Christian Bustamante, Igor Ponomarev, Johann Bauersachs, Denise Hilfiker-Kleiner, Thomas Thum, Evgeni G. Ponimaskin, Melanie Ricke-Hoch

**Affiliations:** 1 Institute of Neurophysiology, Cellular Neurophysiology, Hannover Medical School, Hannover, Germany; 2 Department of Cardiology and Angiology, Hannover Medical School, Hannover, Germany; 3 Institute of Molecular and Translational Therapeutic Strategies, Hannover Medical School, Hannover, Germany; 4 Department of Hematology, Hemostaseology, Oncology and Cell Therapy, Hannover Medical School, Hannover, Germany; 5 Institute of Transplant Immunology, Hannover Medical School, Hannover, Germany; 6 Neuroscience Research Institute, University of California Santa Barbara, Santa Barbara, California USA; 7 Department of Pharmacology and Neuroscience, Texas Tech University Health Sciences Center, Lubbock, Texas USA; 8 Center of Excellence for Translational Neuroscience and Therapeutics, Texas Tech University Health Sciences Center, Lubbock, Texas USA; 9 President, Hannover Medical School, Hannover, Germany

**Keywords:** 5-HT7R, Serotonin, Myocardial infarction, Inflammation, Macrophage

## Abstract

**Background:**

Myocardial infarction (MI) is among the leading causes of death worldwide, and a precisely regulated inflammatory response is essential for effective cardiac repair and long-term outcome. The serotonergic system regulates immune cell functions, yet its contribution to post-MI remodeling is incompletely understood.

**Methods:**

Here, we investigated the role of serotonin receptor 7 (5-HT7R) in post-infarction inflammation and outcome after permanent left anterior coronary artery ligation in mice, and complementary analysis of peripheral blood mononuclear cells (PBMCs) obtained from patients with acute MI.

**Results:**

Three days after MI, 5-HT7R mRNA expression was significantly upregulated in the infarct region compared to remote myocardium and sham operated mice. RNA-sequencing of isolated murine CD11b^+^ cells demonstrated high 5-HT7R expression in cardiac macrophages during the acute phase, which was confirmed by immunohistochemistry. Systemic 5-HT7R-knockout did not affect basal cardiac function but resulted in impaired left ventricular function and enhanced inflammatory signatures 14 days after MI without changes in infarct size. Pharmacological activation of 5-HT7R signaling with the selective agonist LP-211 increased survival after MI, although global systolic function among survivors was not improved. Transcriptomic profiling of cardiac macrophages 3 days after MI revealed bidirectional regulation of inflammatory and metabolic programs depending on 5-HT7R activity, including altered expression of *Got1* and *S100A9*. Complementary, in PBMCs from MI patients *Got1* expression was reduced, while *S100A9* was increased, and both correlated with 5-HT7R expression.

**Conclusions:**

These data suggest 5-HT7R as a regulator of post-MI immune responses and a potential target to improve repair and survival.

**Supplementary information:**

The online version contains supplementary material available at 10.1186/s12967-026-08423-4.

## Background

Myocardial infarction (MI) followed by heart failure (HF) remains one of the leading causes of death worldwide. Despite advances in therapeutic interventions, including percutaneous coronary intervention (PCI) and pharmacological treatments targeting the neurohumoral system, such as β-blockers and angiotensin-converting enzyme (ACE) inhibitors, mortality associated with HF is still high after MI [1, 2]. Following cardiac injury, ischemic cell death initiates a complex cascade of cellular and molecular processes that drive cardiac inflammation, remodeling, and tissue repair [3, 4]. Modulators of the inflammatory response are therefore considered to be effective therapeutic targets to minimize cardiac damage and preserve cardiac function.

Among immune cells, macrophages play a pivotal role in post-infarction remodeling through their dynamic polarization and functional plasticity. However, current therapies are unable to efficiently modulate the inflammation and remodeling processes, thus limiting their impact on long-term cardiac function and survival after MI. Therefore, a deeper understanding of the molecular mechanisms governing inflammation and remodeling is essential for the development of effective interventions.

One potentially promising target is the serotonergic system (5-HT system). Beyond its well-established role in the central nervous system, the 5-HT system regulates the function of various immune cells, and is also affected in various cardiovascular diseases [5, 6]. Notably, patients after acute MI exhibit an elevated risk of developing major depressive disorders (MDD), while patients with MDD or other severe psychiatric disorders are frequently affected by cardiovascular disease associated with increased morbidity and mortality, suggesting a bidirectional link between serotonergic signaling, cardiovascular pathology, and inflammatory responses [7–9]. Our recent data demonstrate that serotonin receptor 7 (5-HT7R) is expressed in human macrophages and modulates their functions [10]. In M1-like macrophages, pharmacological 5-HT7R activation with the highly selective agonist LP-211 reduced phagocytic and migratory ability. In addition, LP-211 treatment altered the secretory profile across all macrophage subtypes indicating that modulation of 5-HT7R activity may represent a novel therapeutic strategy for inflammatory diseases [10].

Based on these findings, we hypothesized that 5-HT7R contributes to the regulation of the inflammatory response after MI and that modulation of the 5-HT7R expression or activity influences cardiac inflammation, remodeling, and survival. In the present study, we therefore investigate the regulation of 5-HT7R signaling in context of MI, aiming to identify if the 5-HT7R is a regulator of post-MI immune responses and a potential target to improve cardiac repair and survival.

## Methods

### Myocardial infarction

Sibling male mice (age 12 to 16 weeks) WT and 5-HT7R-KO (B6;129^SvEv^-Htr7^tm1Sut^, JAX-ID. 019453 [11]), were used in all studies except for time course data (C57BL6/N, purchased from Charles River Germany). Myocardial infarction (MI) was induced by permanent occlusion of the left anterior descending artery (LAD) or sham operation in age-matched mice as described previously [12, 13]. Briefly, one day before surgery, 500 mg/ml metamizol (Ratiopharm) was supplied via the drinking water and maintained until 7 days after surgery. On the day of operation mice received a combination of butorphanol (2 mg/kg body weight (BW), CP Pharma) and carprofen (2 mg/kg BW, CP Pharma) in 0.9% sodium chloride solution (Carl Roth) via subcutaneous injection. Animals were anesthetized with 4% isoflurane (Baxter Deutschland GmbH), intubated and artificially ventilated. Anesthesia was maintained using 1–2% isoflurane. After local disinfection, local anesthesia (0.5% xylocaine, Aspen) was administered prior to the left thoracotomy in the 5^th^ intercostal space. After opening the pericardium, the anterior interventricular branch of the left coronary artery was ligated with a 6.0 Prolene suture (Ethicon). To induce large infarcts, the suture was positioned just below the auricular level. Successful coronary occlusion was indicated by significant color changes in the ischemic region. Mice were randomly assigned to experimental groups. In sham-operated mice, the suture passing around the LAD artery was not tied. Data were collected at baseline and after 3 days, 7 days or 14 days post-MI, as indicated. Mice suffering intraoperative death and mice with an MI size < 20% were excluded from the study.

### LP-211 treatment after MI

Starting on day one after surgery, 0.025 mg/10 g body weight of LP-211 (Sigma Aldrich) in 0.9% sodium chloride solution was injected intraperitoneally (i.p.) once daily until the end of the experiment. Control animals were administered an i.p. injection of 0.9% sodium chloride solution accordingly until the end of the experiment.

### Infarct size measurements

Infarct size measurements were performed on longitudinal myocardial sections stained with Picro-Sirius red F3BA as the ratio of scar length to LV circumference [13]. In brief, hearts were retrogradely perfused (2 min; 80 mmHg) with PBS (pH 7.4) containing 50 mM KCl (Sigma Aldrich/Merck) and 200 U/ml heparin (Ratiopharm), followed by in situ paraformaldehyde-fixation. Hearts were cut longitudinally through the center of the scar, and 6 µm sections were prepared and stained with Picro-Sirius red F3BA (Sigma Aldrich/Merck) [14]. MI sizes were determined in the first three midventricular sections from the midline of the scar, representing the full scar dimensions. Epicardial and endocardial infarct lengths and circumferences were measured, and the epicardial and endocardial infarct ratio was determined by dividing each infarct length by circumference. MI size was calculated as [(epicardial infarct ratio+endocardial infarct ratio)/2] x 100 [15] and is presented as the mean of the three midventricular sections.

### Echocardiography

Transthoracic 2D echocardiography was performed to assess contractile function in 5-HT7R-KO and in corresponding WT mice on anesthetized mice as described previously [16, 17]. In brief, contractile function and heart rate were assessed by serial echocardiography using the Vevo 3100 system (VisualSonics) at baseline and after 3 days or 14 days post-MI (as indicated). Anesthesia was induced with 2% (in 100% oxygen) isoflurane, followed by maintenance at 0.5–1% isoflurane via a special vaporizer for rodents delivered by a small nose cone (VisualSonics). For echocardiographic image acquisition, the animal was placed in a supine position on a prewarmed platform and the body temperature was maintained as close to 37 °C as possible during the entire procedure. Echocardiographic measurements were obtained 5 min following the induction of anesthesia when the heart rate had stably recovered to exclude the variation in cardiac function created by anesthesia induction. Parasternal short- or long-axis views were recorded in B-mode at the level of the papillary muscle, and still images were used to measure left ventricular (LV) end-diastolic diameter (LVEDD) and LV end-systolic diameter (LVESD) and calculate fractional shortening (FS; [LVEDD – LVESD]/LVEDD x 100) in short-axis view and fractional area change (FAC; [end-diastolic area (LVEDA) – end-systolic area (LVESA)/end-diastolic area ×100) in long-axis view.

### Human peripheral blood mononuclear cell (PBMC) isolation

Peripheral blood mononuclear cells (PBMCs) from MI patients (*N* = 18) were isolated from blood taken 3 ± 1d after insult in EDTA containing S-Monovette® tubes (Sarstedt) as described before [18]. Blood from healthy volunteers (*N* = 11) served as control. Blood samples were 1:2 diluted in PBS (Sigma-Aldrich) and loaded on top of 12.5 ml Bicoll Separating Solution (1.077 g/ml, Biochrom). For separation, the tubes were centrifuged for 30 minutes at 800 ×g at room temperature without the brake. The PBMC layer was removed as completely as possible with a pipette, then diluted 1:3 with PBS and centrifuged at 300 ×g for 10 minutes. The supernatant was discarded, and the cells were washed again with PBS. Centrifugation was continued at 200 ×g for another 10 minutes. After isolation, the PBMC cell pellets were directly lysed in TRIzol® reagent (Invitrogen) for RNA isolation and gene expression analysis.

### RNA isolation, cDNA synthesis, qRT-PCR and miR-qRT-PCR

Total RNA from murine hearts was isolated with TRIzol® reagent (Invitrogen) in accordance with the manufacturer’s instructions. cDNA synthesis using Superscript III (Invitrogen), 2 µg of total RNA and random hexamer primers (Sigma-Aldrich) was performed according to the manufacturer’s protocols as previously described [19]. Semi-quantitative real-time PCR using the SYBR green dye method (SYBR Green qPCR 2x Mastermix-Kit, Thermo Fisher Scientific) was performed with the AriaMX Real-Time PCR System (Agilent Technologies). Sequences of qRT-PCR primers used in this study are provided below. mRNA expression levels were normalised using the 2^-ΔΔCT^ method relative to 18S or HPRT.

Expression of mature miR-29a-3p (Applied BioSystems) was determined by miR-qRT-PCR on an ABI7500 cycler (Applied BioSystems) and was normalized using the 2^-ΔΔCT^ method relative to U6 as previously described [20].

#### Sequences of mouse qRT-PCR primers

**Table:** 

mRNA	Sense primers (5’ to 3’)	Antisense primers (5’ to 3’)
mmu *18S*	GTAACCCGTTGAACCCCATT	CCATCCAATCGGTAGTAGCG
mmu *Adgre1*	GAGACATCCACTCTGGGCAC	GGGGCCCCTGTAGATACTGA
mmu *Anp*	GCCGGTAGAAGATGAGGTCA	GGGCTCCAATCCTGTCAATC
mmu *Cd80*	TTTCAGACCGGGGCACATAC	AGAAGCGAGGCTTTGGGAAA
mmu *Cd206*	GATGACCTGTGCTCGAGAGG	TCGCTTCCCTCAAAGTGCAA
mmu *Got1*	GAAGACAATGGCTGACCGGA	TGGATGGAGGTAGCGACGTA
mmu *Hprt*	CATTATGCCGAGGATTTGGAA	TGACATCTCGAGCAAGTCTTTCA
mmu *5-HT7R*	ACCTGAGGACCACCTATCGT	GGCTCCAGTCATGAGCACAAA
mmu *S100a9*	GGACACCCTGACACCCTGA	CTCAAAGCTCAGCTGATTGTCC

#### Sequences of human qRT-PCR primers

**Table:** 

mRNA	Sense primers (5’ to 3’)	Antisense primers (5’ to 3’)
hsa *18S*	AGAACGAAAGTCGGAGGTTCG	GGACATCTAAGGGCATCACAG
hsa *5-HT7R*	CTTCGTCAAGAAGCTCCGCC	TGTGATCCCAAGGTACCTGTCAA
hsa *GOT1*	AGCTGTGCTTCTCGTCTTGC	CACAGCATTGTGATTCTCCCA
hsa *S100a9*	GGGAATTCAAAGAGCTGGTGC	CATTTGTGTCCAGGTCCTCCA

### Macrophage isolation from infarcted mouse hearts

Isolation of neutrophils, macrophages and fibroblasts from basal and infarcted WT hearts after 1d and 7d post-MI or from infarcted 5-HT7R-KO, WT and WT with LP-211 hearts 3 days post-MI was performed after collagenase digestion followed by antibody binding and magnetic-activated cell sorting (MACS) as described previously [19, 21]. In brief, 3 days after MI, LVs were excised and washed with Hanks balanced salt solution (HBSS; Merck). Aortic and pulmonary vessels and atria were removed. Tissues were minced into small pieces and digested for 3 × 15 min at 37 °C with 0.1% collagenase type II (Worthington) and DNase I in HBSS. The digested suspension was filtered through a 30 μm filter. After red blood cell lysis, enrichment of Ly6G^+^ cells (Miltenyi Biotec, 130–092-332) and CD11b^+^ macrophages (Miltenyi Biotec, 130–049-601) was achieved by sorting the cells using microbeads and the MACS system (Miltenyi Biotec). Flow through was collected as CD11b^-^ fibroblasts. Isolated cell fractions were directly lysed in TRIzol® reagent (Invitrogen) for RNA isolation.

### RNA-Seq. (WT vs WT LP-211; WT vs HT7R-KO macrophages)

#### Library generation, quality control, and quantification

100 ng of total RNA per sample were utilized as input for rRNA depletion procedure with ‘NEBNext® rRNA Depletion Kit (Human/Mouse/Rat), 96 rxns’ (New England Biolabs, E6310X) followed by stranded cDNA library generation using ‘NEBNext® Ultra II Directional RNA Library Prep Kit for Illumina’ (New England Biolabs, E7760L). All steps were performed as recommended in user manualE7760 (Version 1.0_02-2017; NEB) except that all reactions were downscaled to 2/3 of initial volumes.

cDNA libraries were barcoded by dual indexing approach, using ‘NEBNext Multiplex Oligos for Illumina − 96 Unique Dual Index Primer Pairs’ (New England Biolabs, 6440S). All generated cDNA libraries were amplified with 10 cycles of final PCR.

One additional purification step was introduced at the end of the standard procedure, using 1.2x ‘Agencourt® AMPure® XP Beads’ (Beckman Coulter Inc., A63881). Fragment length distribution of individual libraries was monitored using ‘Bioanalyzer High Sensitivity DNA Assay’ (Agilent Technologies, 5067–4626). Quantification of libraries was performed by use of the ‘Qubit® dsDNA HS Assay Kit’ (ThermoFisher Scientific, Q32854).

#### Library denaturation and sequencing run

Equal molar amounts of individually barcoded libraries were pooled for a common sequencing run in which each analyzed library constituted around 8.3% of overall flowcell / run capacity. The library pool was denatured with NaOH and was finally diluted to 1.8 pM according to the Denature and Dilute Libraries Guide (Document # 15048776 v02; Illumina). 1.3 ml of the denatured pool was loaded on an Illumina NextSeq 550 sequencer using a High Output Flowcell (400 M cluster) for single reads (Illumina, 20024906). Sequencing was performed with the following settings: Sequence reads 1 and 2 with 38 bases each; Index reads 1 and 2 with 8 bases each.

#### BCL to FASTQ conversion

BCL files were converted to FASTQ files using bcl2fastq Conversion Software version v2.20.0.422 (Illumina).

#### Raw data processing and quality control

Before downstream analysis, all FastQ files were quality-checked and filtered using fastp v0.23.4 [22]. The parameter -q 30 was used to filter out bases with a Phred score lower than 30. All other settings were run with default parameters. After quality control and quantification, the read depth for all samples ranged between 14 and 22 million reads, with an average of 17 million reads.

Salmon v1.10.2 was used to quantify transcript expression [23]. GRCm39 was used as the reference genome to build a decoy-aware index. This genome was obtained from Ensembl release 112 [24]. Once the index was built, the quant command was run with the -l A and –validateMappings arguments. To generate a matrix of raw counts, the output from salmon was imported to R and summarized to gene level using the package Tximeta 1.24.0 [25]. A Principal Component Analysis (PCA) was run on log2 transformed counts to visualize the primary sources of variation and potential outliers. PCA plots were generated with ggfortify [26].

#### Normalization and differential expression analysis

Differentially expressed genes (DEGs) were identified using edgeR v4.4.2 [27] and annotated with AnnotationDbi v1.68.0 and org.Mm.eg.db v3.20.0 [28, 29]. Prior to differential expression analysis, normalization by library size was performed using the edgeR functions calcNormFactors and normLibSizes, and low-expressed genes were removed using filterByExpr. Pairwise comparisons were then performed among all three groups. Volcano plots and Venn diagrams were generated using ggplot2, ggrepel, and ggvenn [30–32]. Heatmaps showing normalized gene expression were generated using the ComplexHeatmap package [33].

#### Biological process and pathway overrepresentation

Over-representation analysis (ORA) of biological processes and pathways was conducted using ClusterProfiler v4.14.6 with the Gene Ontology (GO), Reactome, and Kyoto Encyclopedia of Genes and Genomes (KEGG) databases [34–37]. Genes with a nominal *p*-value < 0.05 were used as queries, and all detected genes were used as the background set. Upregulated and downregulated genes were analyzed separately. To identify connections between overrepresented terms, gene networks were constructed using the cnetplot function from the Enrichplot package [38]. Dot plots showing overrepresented terms were also generated using Enrichplot.

### Multiplex assays of cardiac murine tissue

For multiplex analysis, samples from the infarct region of 5-HT7R-KO and WT hearts 3 days post-MI were isolated with the Bio-Plex® cell lysis kit (Bio-Rad, 171304011) according to the manufacturer’s protocol. The protein concentration of the lysates was measured with Bradford reagent (Bio-Rad) and LV lysates were diluted with Bio-Plex sample diluent to a protein contration of 1 mg/ml. The Bio-Plex Pro™ Mouse Cytokine 23-plex Assay (Bio-Rad, M60009RDPD) was used as recommended by the manufacturer and measured with the Bio-Plex 200 system (Bio-Rad).

### Histology and immunostaining

Histological, immunohistological and morphological analyses were performed 3 days and 14 days after sham operation or MI. For cardiac morphological analyses, hearts were embedded in Tissue-Tek O.C.T Compound (Sakura Finetek) and frozen at −80 °C until sectioning. Subsequently, 6 µm cryosections were stained with hematoxylin and eosin (H&E), wheat germ agglutinin (WGA, Vector Laboratories Inc., RL-1022) or IB4 (isolectin B4)-fluorescein (Vector Laboratories Inc., B-1205) as described previously [12]. The cross-sectional area was determined on longitudinal cryosections after wheat germ agglutinin/Hoechst staining. Analysis was done on maximum-intensity projcetions of five centred planes using “HeartJ” plugin in ImagJ with minor modifications [39].

To analyze cardiac 5-HT7R levels, immune cells status and angiogenesis, 6 µm LV cardiac slices were stained with respective antibodies. In brief, cardiac slices were washed with PBS, blocked and permeabilized using 10% donkey serum (Jackson ImmunoResearch) and 0.3% Triton X-100 (Sigma Aldrich) for 45 min. The slices were stained overnight using primary antibodies recognizing CD68 (Novusbio, NBP2-33337), CD80 (Abcam, ab106162), CD206 (R&D Systems, AF2535), CD31 (eBioscience™, Invitrogen, 14–0311-82), Nestin (ThermoFisher, PA5-143578), and 5-HT7R (BioRad, AHP1343) at 4 °C. Following secondary antibodies were used: Alexa Fluor® 488 AffiniPure™ Goat Anti-Rabbit IgG (H+L) (Jackson ImmunoResearch, 111–545-003), Alexa Fluor® 546 Goat anti-Rat IgG (H+L) (Dianova, A-11081), Alexa Fluor® 594 AffiniPure™ Donkey Anti-Goat IgG (H+L) (Jackson ImmunoResearch, 705–585-147), Alexa Fluor® 647 AffiniPure™ Goat Anti-Armenian Hamster IgG (H+L) (Jackson ImmunoResearch, 127–605-160), Alexa Fluor® 647 AffiniPure Donkey Anti-Chicken(Jackson ImmunoResearch, 703–605-155), DyLight® 649 Donkey Anti-Rat IgG (H+L) (Jackson ImmunoResearch, 712–495-153), Alexa Fluor® 488 Anti-Mouse (Jackson ImmunoResearch, 715–545-150), Cy3 Anti-Rabbit (Jackson ImmunoResearch, 711–165-152) and counterstained with Hoechst 33342 trihydrochloride, trihydrate (Invitrogen) or 4′,6-Diamidino-2-phenylindol (DAPI; Sigma Aldrich).

The tissue was imaged on a Zeiss LSM780 with a LD C-Apochromat with a 40x/1.2 W objective and Zen 2012 imaging software. Each fluorophore/dye was imaged in online-fingerprinting mode with pre-defined spectra of single-stained slices. The evaluation was conducted blinded on maximum-intensity projections applying custom-written Matlab scripts. In each cardiac slices two regions of the scar, border, and remote area were captured with a pixel size of 0.208 µm (1024 × 1024 pixels; 212.55 × 212.55 µm), pixel dwell time 1.57 µs. Manders correlation coefficient was determined using a custom-written Matlab script and averaged on four regions per animal.

### Statistical analyses

Database management and statistical analyses were performed with the PRISM version 7.0 and version 11.0 statistical programme (GraphPad Software Inc., La Jolla). Data are presented as mean ± SD. Differences between groups were analysed by Student’s *t*-test with or without Welch’s correction or ANOVA with Bonferroni post hoc tests. A non-parametric Mann-Whitney test was performed on data not fitting a Gaussian distribution (as analysed by D’Agostino and Pearson omnibus normality test or Shapiro-Wilk normality test for small sample sizes). A *P* value of <0.05 was considered statistically significant. Data are depicted as *p < 0.05, **p < 0.01, ***p < 0.001, ****p < 0.0001.

## Results

### 5-HT7R is predominantly expressed in inflammatory cells in response to myocardial infarction in the adult heart

**Fig. 1:**
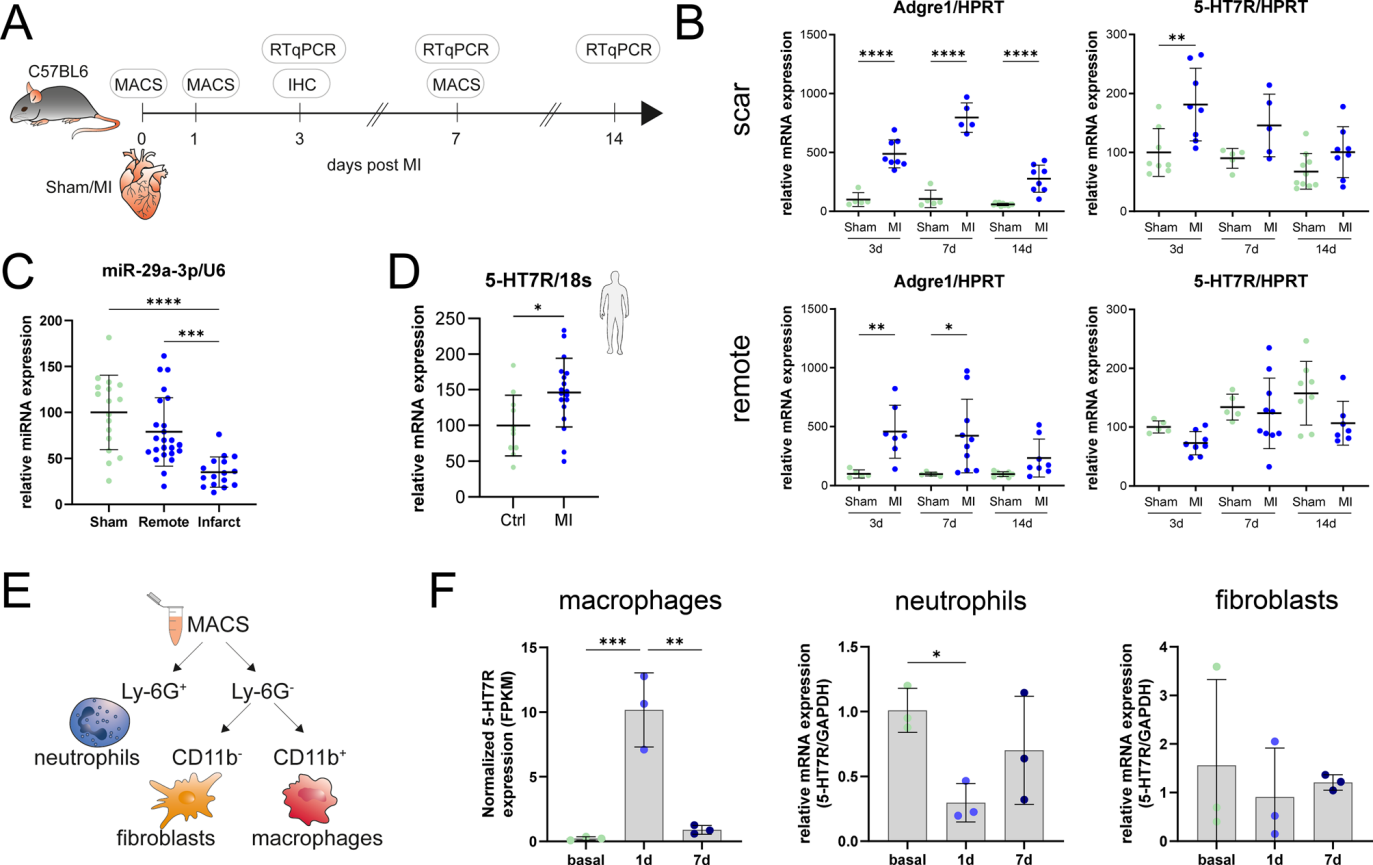
Increased expression of 5-HT7R post-MI in mouse and human. (**A**) Graphical illustration of mouse MI experiments. C57BL6 mice received either sham or MI surgery. Cardiac tissue was collected on 0, 1, 3, 7 and 14 days (d) post-MI and was further processed either by magnetic-activated cell sorting (MACS) separation technology or used for immune histochemistry (IHC) and quantitative realtime PCR (RTqPCR). (**B**) Quantification of mRNA expression levels of adhesion G protein-coupled receptor E1 (Adgre1) and 5-HT7R normalized to hypoxanthine-guanine phosphoribosyltransferasein (HPRT) in cardiac scar and remote regions 3, 7, and 14 days after MI in animals receiving sham or MI-operation (3d Sham, 7d Sham, 7d MI: *N* = 5; 3d MI, 14d MI: *N* = 8; 14d Sham: *N* = 11). (**C**) Dot plot showing miR-29a-3p levels (mean value) normalized to U6 of LV tissue from remote myocardium (*N* = 24) and infarct area (*N* = 16) from mice 3 days post-MI compared to LV tissue from sham-operated mice (*N* = 17). (**D**) Relative mRNA expression of 5-HT7R in peripheral blood mononuclear cells (PBMCs) of MI patients (*N* = 18) 3 ± 1d after insult compared to healthy controls (*N* = 11) normalized to 18S rRNA. (**E**) Experimental scheme of isolation of neutrophils, fibroblasts, and macrophages by MACS separation technique. (**F**) 5-HT7R expression in macrophages, neutrophils and fibroblasts basal, 1 and 7 days post-MI (*N* = 3). Macrophage 5-HT7R expression is normalized to fragments per kilobase of transcript per million mapped reads (FPKM), whereas neutrophil and fibroblast expression is shown relative to GAPDH expression. Statistical analysis was performed using (**B**) ordinary One-Way ANOVA with Šídák multiple comparison post hoc test, (**C**) One-Way ANOVA followed by post hoc tests with Bonferroni’s correction (**D**) unpaired, two-tailed *t*-test and (**F**) ordinary One-Way ANOVA with tukey multiple comparison post hoc test

To investigate whether the 5-HT7R expression is regulated in the heart after MI, we quantified 5-HT7R mRNA levels in the infarcted (scar) area and in the remote myocardium 3, 7 and 14 days post-MI and compared them with LVs from sham-operated WT mice (Fig. 1A, B). Upregulation of cardiac stress and hypertrophy marker *atrial natriuretic peptide* (*ANP*) together with increased expression of inflammatory markers, including the macrophage-associated genes *Adgre1*, *CD80* and *CD206*, confirmed effective cardiac injury and initiation of inflammatory and remodeling processes (Fig. 1B; Supplementary Fig. S1A-C). Notably, 5-HT7R mRNA expression was significantly increased exclusively in the infarcted (scar) region during the acute phase 3 days post-MI (Fig. 1B). This time point coincides with the peak of inflammatory activity after MI before the beginning transition from M1 to M2 macrophage polarisation [4]. To explore potential post-transcriptional mechanisms underlying this transient upregulation, we performed miRNA target prediction analysis using TargetScanMouse 7.2, which identified miR-29a-3p as a putative regulator of 5-HT7R expression. Consistent with increased 5-HT7R mRNA levels, miR-29a-3p expression was markedly reduced in the infarct region compared to the remote myocardium 3 days after MI, and compared to the LV of sham-operated controls (Fig. 1C), suggesting miRNA-mediated regulation of 5-HT7R expression in response to cardiac injury.

To assess clinical relevance, we analyzed peripheral blood mononuclear cells (PBMCs) from patients 3 ± 1 days after acute MI. PBMCs from MI patients exhibited significantly higher 5-HT7R mRNA expression compared with healthy controls (Fig. 1D), supporting translational relevance of the observed regulation.

To further characterize cell-specific expression patterns, Ly-6 G^+^ neutrophils, CD11b^-^ fibroblast and CD11b^+^ macrophages were isolated from mouse hearts under basal conditions and at 1 and 7 days post-MI using magnetic-activated cell sorting (MACS) separation (Fig. 1E). RNA-sequencing (RNA-Seq) analysis from CD11b^+^ macrophages revealed a pronounced increase in 5-HT7R expression at 1 day post-MI, whereas Ly-6 G^+^ neutrophils showed reduced 5-HT7R expression at this timepoint. In contrast, the 5-HT7R expression in CD11b^-^ fibroblasts remained unchanged (Fig. 1F). Moreover, expression of 5-HT7R-related signaling partner *Cd44* was significantly upregulated in macrophages 1 day after MI, whereas expression of others downstream signaling molecules including *Cdc42*, *Mmp9*, *RhoA*, *G*α*S* and *G*α*12* was not significantly altered (Supplementary Fig. S1D).

**Fig. 2:**
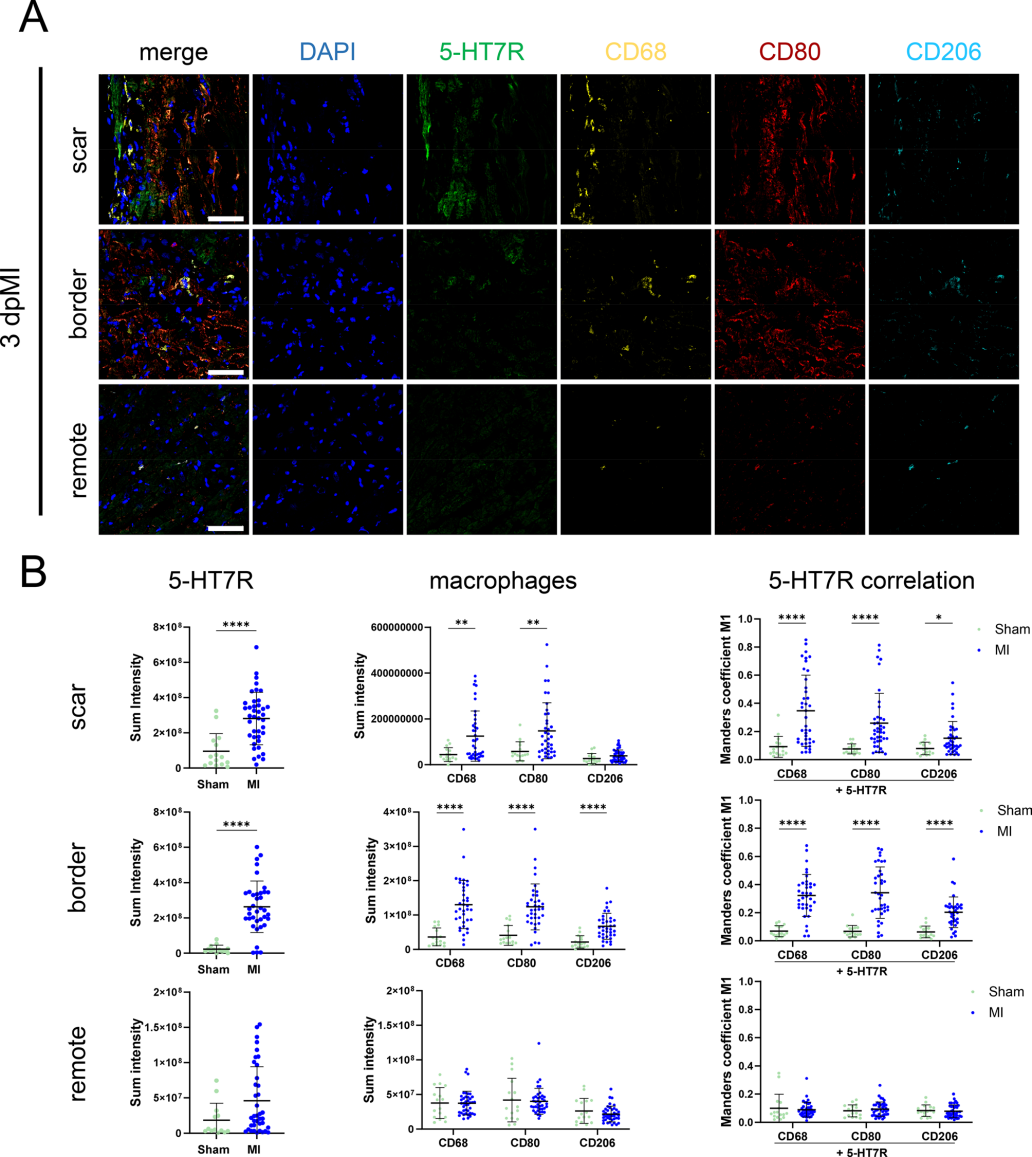
5-HT7R expression in murine macrophages 3 days post-MI. (**A**) Images of scar, border, and remote cardiac sections post sham or MI surgery after 3 days. 5-HT7R (green), CD68 (yellow), CD206 (turquoise), CD80 (red) and nuclei (DAPI; blue) are visualized. Scale bars 50 μm. (**B**) Quantification of the sum intensity of 5-HT7R and macrophage markers. The Manders coefficient was assessed between 5-HT7R and CD68, CD206, and CD80 macrophage marker signals, respectively, in scar, border, and remote cardiac slices 3 days post-MI compared with sham-operated animals (Sham: *n* = 16 slices from 4 animals; MI: *n* = 40 slices from 10 animals). Mann-Whitney with Holm-Šídák multiple comparison post hoc test was used for statistical analysis

Immunofluorescence staining for 5-HT7R in combination with the macrophage markers CD68, CD80 and CD206 on infarcted WT hearts confirmed robust expression of 5-HT7R in macrophages within the scar and border zone, which was supported by positive Mander’s correlation coefficient. In contrast, the remote myocardium showed only low signal intensity for all four markers, indicating minimal expression outside the infarcted area (Fig. 2A, B).

### 5-HT7R deficiency impairs cardiac function 14 days post-MI, while pharmacological 5-HT7R activation improves survival

To address the functional relevance of 5-HT7R signaling for cardiac function and outcome after MI, systemic 5-HT7R-knockout (KO) mice and corresponding wildtype (WT) littermates were analyzed after 3 days and 14 days post-MI. Baseline echocardiographic analysis revealed that 5-HT7R-KO mice at the age of 3–4 months show normal cardiac function compared to WT mice (Supplementary Table S1). Moreover, heart weight (HW), body weight (BW), lung weight (LW) and the HW/BW or LW/BW ratio did not differ between genotypes under basal conditions (Table 1).

**Table 1:** Cardiac function and dimensions in male 5-HT7R-KO and WT mice 14 days after MI. Echocardiographic analysis was performed in 14–16 weeks old male 5-HT7R-KO and corresponding WT mice. Data are depicted as mean ± SD or as median with IQR depending on gaussian distribution, *p < 0.05 vs WT MI, statistical analysis was done using unpaired *t*-test (FAC, LVEDA, LVESA, HR, HW, BW, HW/BW) and Mann Whitney test (LW wet, LW dry, LW/BW), respectively

Parameters	WT sham *N* = 5	5-HT7R-KO sham *N* = 5	WT MI *N* = 12	5-HT7R-KO MI *N* = 11	WT MI LP-211 *N* = 12
FAC [%]	48.1 ± 8.0	49.7 ± 8.4	17.2 ± 6.5	11.2 ± 3.1*	13.7 ± 4.7
LVEDA [cm^2^]	0.225 ± 0.021	0.219 ± 0.013	0.388 ± 0.084	0.449 ± 0.107	0.385 ± 0.047
LVESA [cm^2^]	0.117 ± 0.020	0.111 ± 0.022	0.325 ± 0.091	0.400 ± 0.105	0.333 ± 0.049
HR [bpm]	633 ± 18	607 ± 18	605 ± 26	598 ± 37	609 ± 46
	*N* = 5	*N* = 5	*N* = 9	*N* = 9	*N* = 9
HW [mg]	132.2 ± 15.0	126.2 ± 10.7	154.0 ± 22.9	168.1 ± 26.2	147.3 ± 16.6
BW [mg]	26.0 ± 2.0	25.0 ± 2.7	25.9 ± 1.9	26.04 ± 2.0	25.6 ± 1.4
HW/BW [mg/mg]	5.09 ± 0.48	5.07 ± 0.38	5.94 ± 0.79	6.50 ± 0.13	5.76 ± 0.69
LW wet [mg]	142.1 (138.4–150.1)	144.1 (140.6–254.5)	145.8 (129.5–153.9)	174.2 (140.9–203.4)	134.6 (126.3–147.4)
LW dry [mg]	35.6 (35.6–36-6)	33.9 (33.4–54.83)	33.7 (30.4–39.3)	39.7 (33.3–46.8)	32.3 (29.8–35.4)
LW/BW [mg/mg]	5.48 (5.04–6.12)	5.51 (5.30–11.64)	5.72 (5.09–6.22)	6.41 (5.45–7.84)	5.21 (4.94–5.73)

FAC, fractional area change; LVEDA, left ventricular enddiastolic area; LVESA, left ventricular endsystolic area; HR, heart rate (bpm, beats per minute) determined in parasternal long-axis (PSLAX) view recorded in B-mode; HW, heart weight; BW, body weight; LW, lung weight

At 3 days after MI, echocardiography revealed comparable LV dimensions and systolic function in 5-HT7R-KO and WT mice (Supplementary Table S2). However, assessment of the cardiac inflammatory milieu by Bioplex analysis revealed elevated baseline chemokine and cytokine levels in sham-operated 5-HT7R-KO mice compared with WT controls, including IL-1a, IL-1b, IL-3, IL-5, IL-6, IL-10, IL-12, IL-13, IL-17, GM-CSF, CXCL1, MCP-1, MIP-1b, TNF-a. 3 days following MI, 5-HT7R-KO hearts experienced a more pronounced increase in IL-1a, IL-5, and CXCL1 levels compared to WT mice (Supplementary Fig. S2), indicating an enhanced inflammatory response in the absence of 5-HT7R signaling.

**Fig. 3:**
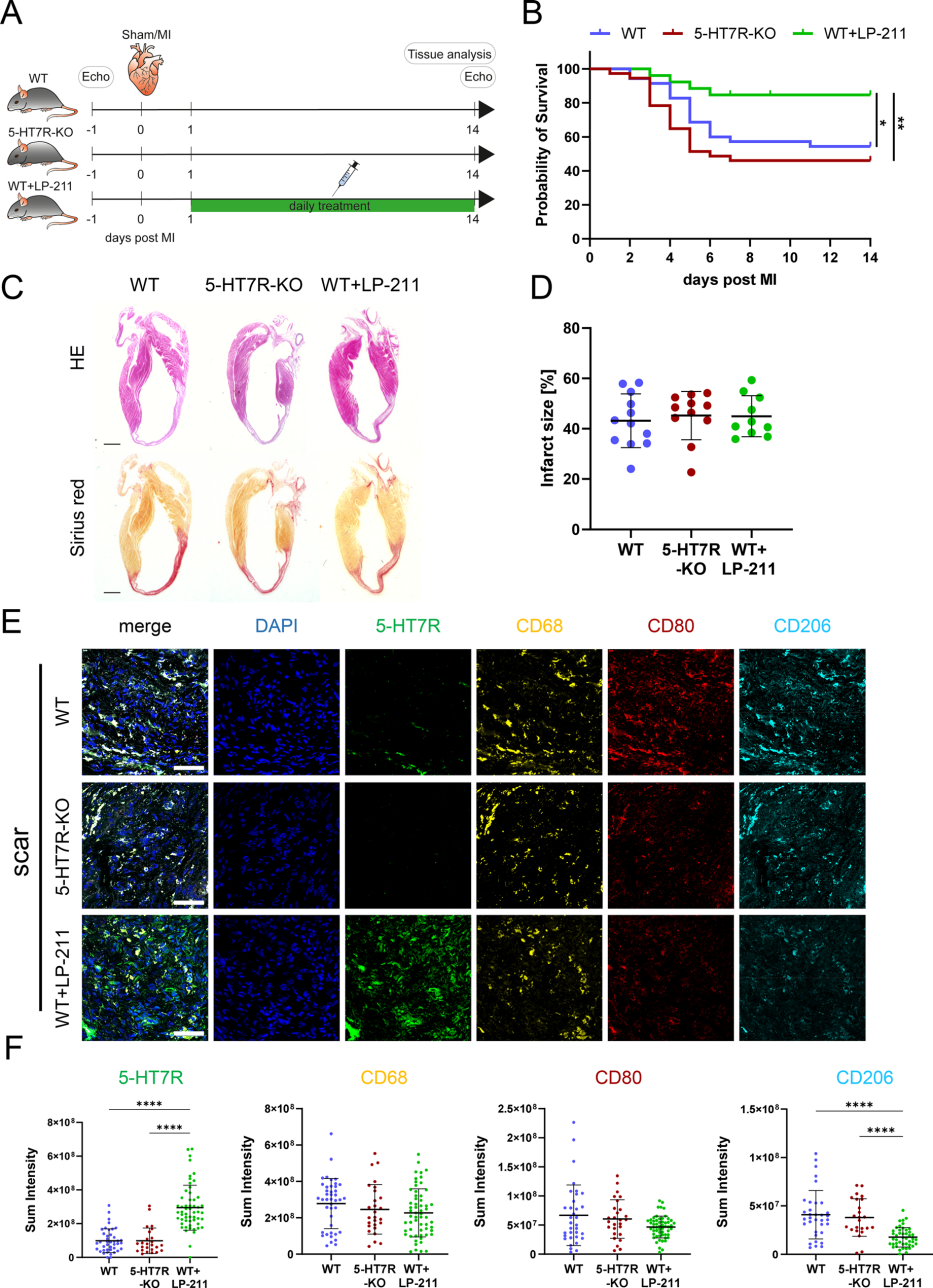
LP-211 treatment increases survival and reduces macrophage infiltration into the heart. (**A**) Graphical time line of treatment regime during MI experiments. C57BL/6J WT mice and 5-HT7R-KO mice were subjected to echocardiography (Echo) one day prior surgery. The mice received either sham or MI surgery. One day after surgery, WT animals received a daily injection of LP-211. On day 14 a final echocardiography was performed followed by cardiac tissue collection. (**B**) Kaplan-Meier analysis of WT (*N* = 35), 5-HT7R-KO (*N* = 37) and WT treated with LP-211 (WT+LP-211) (*N* = 26) upon 14 days post operation. (**C**) Representative images of hematoxylin and eosin (HE) and Sirius red stained cardiac slices 14 days post-MI. Scale bars 2 mm. (**D**) Dot plot showing infarct sizes in WT, 5-HT7R-KO, and WT+LP-211 mice (WT *N* = 12; 5-HT7R-KO *N* = 11; WT+LP-211 *N* = 10) 14 days after MI. (**E**) Representative images of cardiac scar regions visualizing 5-HT7R (green) and macrophage marker CD68 (yellow), CD80 (red), and CD206 (turquoise) and nuclei (DAPI; blue) expression 14 days after surgery. Scale bars 50 µm. (**F**) Quantification of the sum intensity of every analyzed marker in the scar region 14 days post-MI in WT, 5-HT7R-KO and WT+LP-211 animals (WT: *n* = 40 slices from 10 animals; 5-HT7R-KO: *n* = 28 slices from 5 animals; WT+LP-211: *n* = 60 slices from 15 animals). Statistical analysis was performed using (**B**) Log-rank (Mantel-Cox) test at 14 days post-MI, (**D**) Kruskal-Wallis test with Dunn’s multiple comparison post-hoc test, and (**F**) Kruskal-Wallis with Dunn’s multiple comparison test

Interestingly, 14 days post-MI, 5-HT7R-KO mice displayed a significantly greater decline in LV function and increased markers of tissue inflammation compared with WT mice, while the survival rates and infarct size did not differ between genotypes (Fig. 3A–F, Table 1). Sham-operated 5-HT7R-KO and WT mice showed comparable cardiac function at this time point, confirming that the observed functional impairment was MI-dependent. These findings provide first evidence that endogenous 5-HT7R signaling is important for cardiac integrity and cardiac function during post-infarction remodeling.

In line with these observations, systemic administration of the selective 5-HT7R agonist LP-211 significantly improved survival of WT mice, assessed at 14 days post-MI, although global cardiac function was not improved (Fig. 3A–B, Table 1). This data suggests that LP-211 may confer survival benefits through modulation of inflammatory signaling of immune cells, rather than directly contributing to improved systolic performance. Indeed, LP-211-treated WT mice (WT+LP-211) presented increased 5-HT7R expression and reduced cumulative CD206^+^ macrophage signal intensity in scar regions, border zone and remote myocardium, indicating that 5-HT7R signaling influences macrophage populations and inflammatory signaling in the infarcted myocardium (Fig. 3E–F; Supplementary Fig. S3A-D).

### Modulation of 5-HT7R activity after MI influences cardiac angiogenesis but does not affect cardiomyocyte size or scar maturation

To investigate whether modulation of 5-HT7R signaling affects post-infarction tissue remodeling, cardiac angiogenesis and fibrosis development were analysed 14 days after MI in hearts from WT, 5-HT7R-KO and WT mice treated with the selective 5-HT7R agonist LP-211 (WT+LP-211) (Supplementary Fig. S4, Supplementary Fig. S5). Quantitative immunofluorescence analysis of endothelial markers revealed that 5-HT7R-KO mice displayed an angiogenic response in the infarct border zone comparable to WT mice. In contrast, LP-211-treated animals revealed a significantly increased capillarization in the border zone, as indicated by increased mean fluorescence intensity of the endothelial marker CD31 and active angiogenesis marker nestin (Supplementary Fig. S4A-B), suggesting that pharmacological activation of 5-HT7R promotes post-infarction neovascularization.

To assess whether altered 5-HT7R signaling influences cardiomyocyte hypertrophy or vascular density in non-injured myocardium, cardiomyocyte cross-sectional area and capillary density were analyzed in the remote myocardium. No significant differences were observed among WT, 5-HT7R-KO, and WT+LP-211 groups, indicating that neither genetic ablation nor pharmacological activation of 5-HT7R affects cardiomyocyte size or baseline vascular architecture outside the infarcted area (Supplementary Fig. S5A-B).

In addition, cardiac remodeling was evaluated by Sirius red polarization microscopy to quantify collagen deposition and maturation within the scar and border zone. Total collagen content within the scar was comparable across all experimental groups. Detailed analysis of collagen fiber composition revealed no differences in the ratio of thick (orange-red) to thin (yellow-green) birefringent collagen fibers between WT, 5-HT7R-KO, and WT+LP-211 mice (Supplementary Fig. S5C-F), indicating that 5-HT7R signaling does not significantly influence collagen organization or scar maturation at this stage after MI.

Collectively, these data demonstrate that pharmacological activation of 5-HT7R enhances neoangiogenesis in the infarct border zone after myocardial infarction, as evidenced by increased endothelial marker expression, without affecting cardiomyocyte size, capillary density in remote myocardium, or scar composition.

### 5-HT7R activation influences the molecular phenotype of macrophages 3 days after MI

**Fig. 4:**
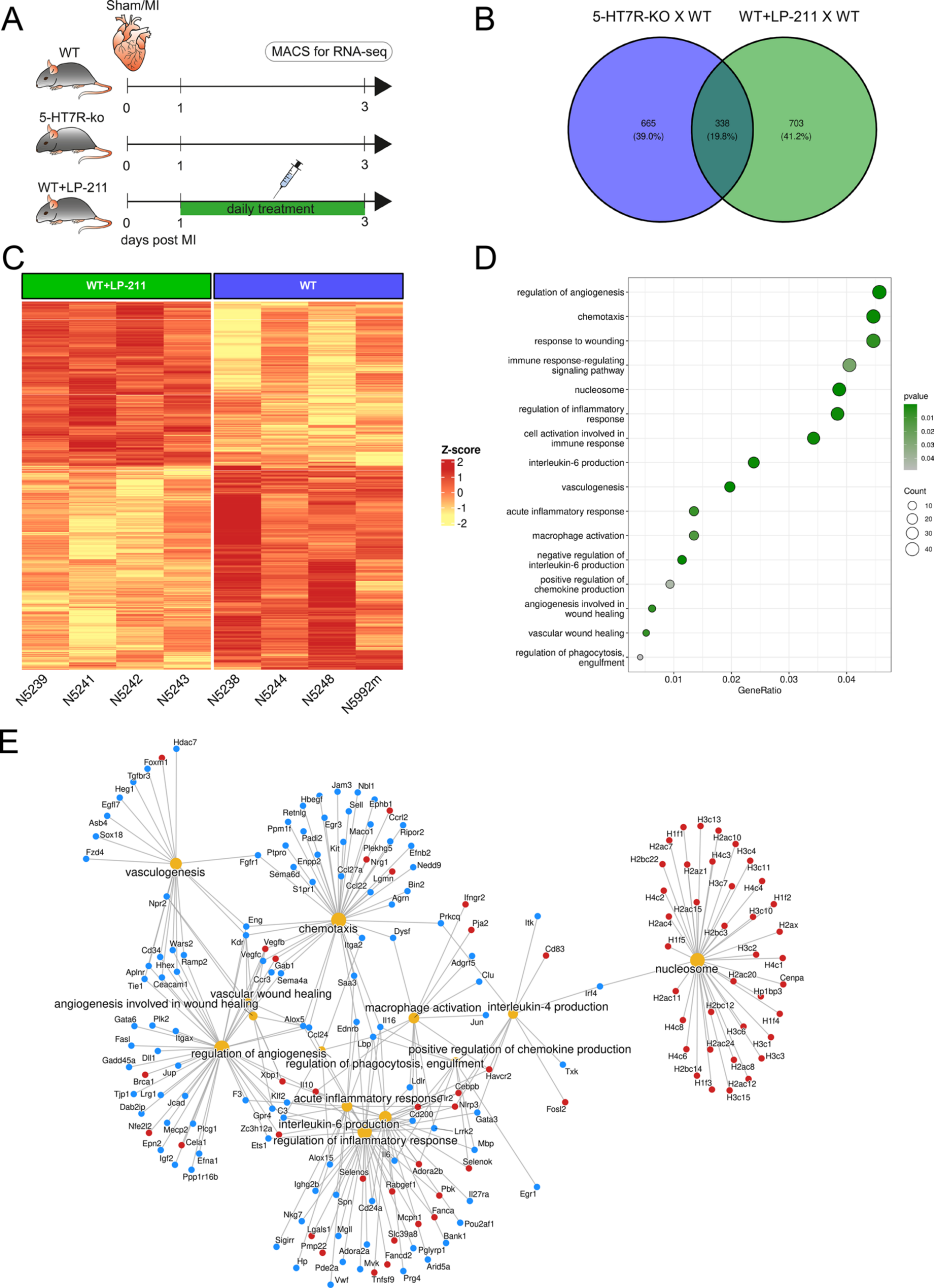
Inflammatory profile of macrophages 3 days post-MI. (**A**) Graphical illustration of experimental setup. The mice underwent echocardiography one day prior surgery. Starting one day after surgery, WT animals received a daily injection of LP-211. On day 3 after surgery macrophages were isolated and subjected to RNA sequencing. (**B**) Venn diagram comparing differently expressed genes between 5-HT7R-KO X WT and WT+LP-211 X WT. (**C**) Heatmap indicates differently expressed genes between macrophages of WT and LP-211-treated WT mice. Each column dataset corresponds to one animal. (**D**) Dotplot of Gene ontology (GO) and Kyoto Encyclopedia of Genes and Genomes (KEGG) enrichment analysis showing important pathways for immune regulation and healing processes following MI, plotted against the gene ratio. The size of the dots represents the count of differentially expressed genes within the pathway and the colour the *p*-value. (**E**) Gene enrichment analysis of differentially expressed genes in WT+LP-211 compared to WT macrophages 3 days after MI. Pathways are indicated in yellow. Upregulated genes are shown in red and downregulated genes in blue

To investigate underlying molecular pathways, Ly6G^-^ CD11b^+^ macrophages were isolated from infarcted mouse hearts 3 days post-MI followed by RNA-Seq and comprehensive pathway analysis (Fig. 4A–E; Supplementary Fig. S6). Comparative transcriptomic profiling revealed substantial changes in gene expression upon genetic ablation or pharmacological activation of 5-HT7R. Specifically, 1003 genes were differentially expressed in macrophages from 5-HT7R-KO mice compared with WT controls, whereas 1041 transcripts were significantly regulated in macrophages from WT mice treated with the selective 5-HT7R agonist LP-211 relative to WT macrophages (Fig. 4B, C). Notably, 338 genes (19.8% of all regulated transcripts) were shared between both comparisons, which likely represent key downstream targets of 5-HT7R signaling thus highlighting the regulatory role of the receptor in shaping macrophage responses during the early post-infarction phase (Fig. 4B).

Gene ontology (GO) and Kyoto Encyclopedia of Genes and Genomes (KEGG) pathway analysis revealed that modulation of 5-HT7R activity predominantly affected transcriptional programs associated with inflammatory signaling, immune regulation and wound healing processes (Fig. 4D, E, Supplementary Fig. S6D). Gene enrichment analysis further revealed the complex and coordinated regulaton of inflammatory pathway networks in macrophages both upon LP-211-mediated receptor activation and in absence of 5-HT7R siganling, underscoring the role of this receptor in post-MI macrophage biology (Fig. 4E, Supplementary Fig. S6E).

**Fig. 5:**
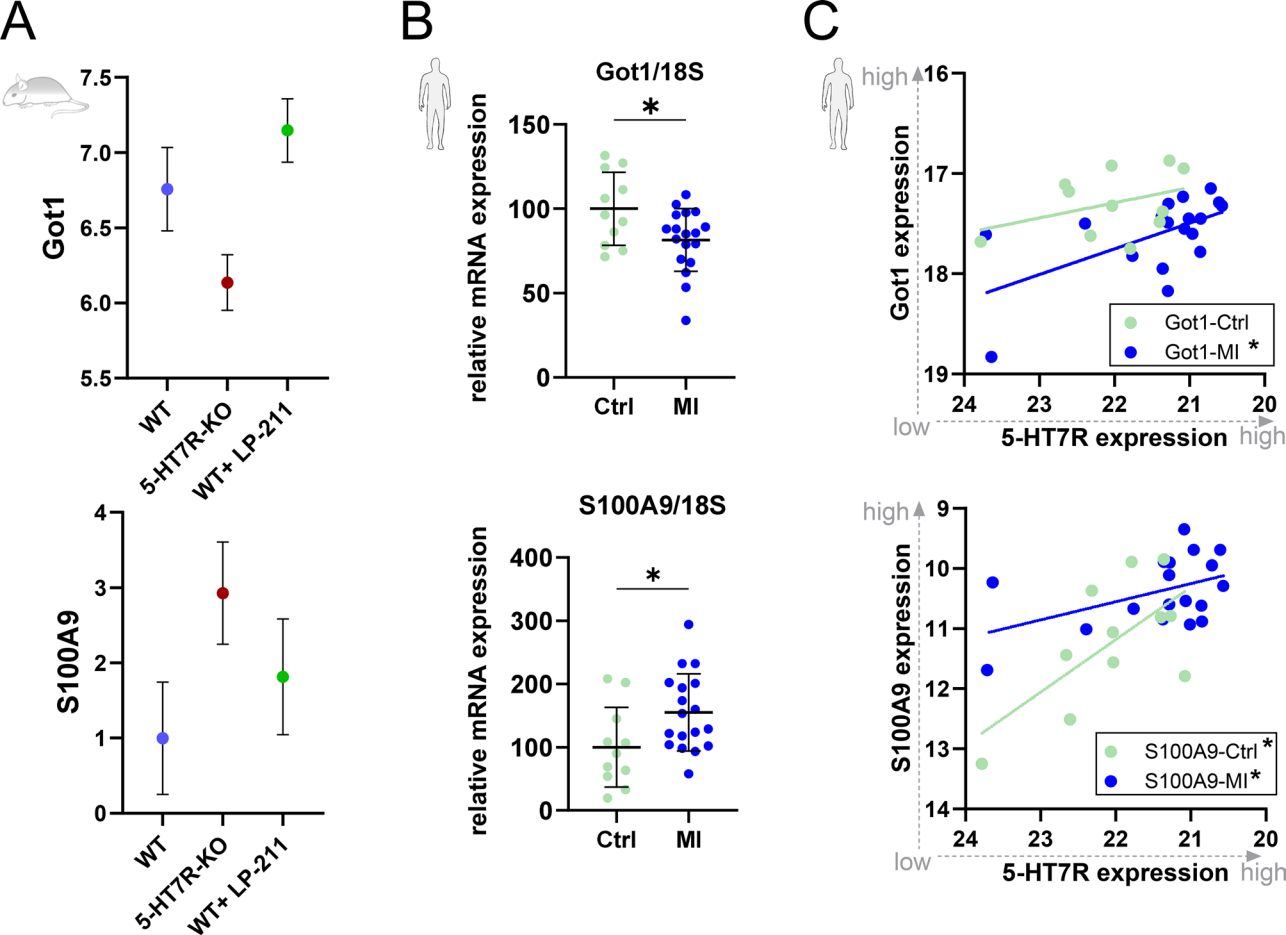
Regulation of Got1 and S100A9 expression in inflammatory cells 3 days after MI. (**A**) mRNA expression of Glutamic-Oxaloacetic Transaminase 1 (Got1) and S100 Calcium Binding protein A9 (S100A9) in macrophages isolated from the mouse heart 3 days post-MI, determined by RNA-sequencing. (**B**) Relative mRNA expression of Got1 and S100A9 in peripheral blood mononuclear cells (PBMCs) of MI patients (*N* = 18) 3 ± 1d after insult compared to healthy controls (*N* = 11) normalized to 18S rRNA. (**C**) Correlation analysis of Got1 and S100A9 expression against 5-HT7R expression in human PBMCs. Axis values show ΔCT values normalized to 18S, so lower numbers indicate greater expression levels. Simple linear regression and correlation analysis revealed positive correlation for 5-HT7R expression and Got1 in PBMCs of patients 3 days after MI (R^2^ = 0.3345; *p* = 0.0104) and for 5-HT7R expression and S100A9 in Ctrl subjects (R^2^ = 0.4338; *p* = 0.0275) and MI patients (R^2^ = 0.2235; *p* = 0.0476)

Among the differentially regulated genes, two particularly interesting gene candidates were identified, namely S100 Calcium Binding Protein A9 (*S100A9*) and Glutamic-Oxaloacetic Transaminase 1 (*Got1*), both of which are known to be involved in post-MI inflammatory and metabolic responses, but have not previously been linked to serotonergic signaling. We found that expression of *Got1* was significantly reduced in macrophages from 5-HT7R-KO mice compared with WT macrophages, whereas macrophages from LP-211-treated WT mice exhibited increased *Got1* expression relative to WT controls (Fig. 5A). In contrast, *S100A9* expression was higher in macrophages from 5-HT7R-KO mice compared to WT macrophages, while the observed elevation through LP-211 treatment did not reach statistical significance. Such bidirectional regulation of inflammatory and metabolic genes, including *Got1* and *S100A9*, suggests that 5-HT7R signaling can be involved in controlling macrophage-driven inflammatory responses and early remodeling processes in the infarcted myocardium.

### Regulation of *Got1* and *S100A9* expression in human inflammatory cells 3 days after MI

To assess the translational relevance of the identified target genes in the human system, we analyzed expression levels of *Got1* and *S100A9* in PBMCs isolated from patients 3 ± 1 days after acute MI. These PBMCs have previously been shown to exhibit significantly increased 5-HT7R expression after MI (Fig. 1D). In line with the murine data, PBMCs from MI patients displayed significantly reduced *Got1* mRNA expression and concomitantly increased *S100A9* expression compared with healthy control individuals (Fig. 5B). Importantly, expression levels of both *Got1* and *S100A9* showed a positive correlation with 5-HT7R mRNA expression, with this association being particularly pronounced in PBMCs from MI patients (Fig. 5C). These findings support the clinical relevance of *Got1* and *S100A9* as downstream targets of 5-HT7R signaling.

## Discussion

Current research focuses on identifying novel molecular targets to improve patient outcomes after MI. In this context, the serotonergic (5-HT) system has emerged as an attractive candidate [5].

In the present study, we identify 5-HT7R as a relevant modulator of the inflammatory response after MI. We demonstrated that 5-HT7R expression is transiently upregulated during the acute phase after MI both in human PBMCs and in mouse hearts. The modulation of 5-HT7R appears to be regulated post-transcriptionally via the expression of miRNAs. Importantly, this upregulation is mainly confined to inflammatory cells, in particular macrophages located in the infarct and border regions of the heart, suggesting that 5-HT7R signaling may be specifically involved in immune cell-mediated processes during early post-infarction remodeling.

To assess the functional relevance of 5-HT7R signaling in vivo, we combined genetic and pharmacological approaches. Systemic deletion of 5-HT7R did not affect cardiac function under basal conditions but resulted in significantly impaired left ventricular function and increased inflammatory signatures 14 days after MI, despite unchanged infarct size. These findings indicate that 5-HT7R signaling is dispensable for normal cardiac homeostasis but becomes critical during post-infarction repair. Conversely, pharmacological activation of 5-HT7R using the selective agonist LP-211 [40, 41] significantly improved survival after MI. Of note, no significant differences in cardiac function were detected between WT mice treated with LP-211 and untreated WT controls. This lack of functional differences might be explained by survivor bias, as animals with severely impaired cardiac function predominantly died during the subacute phase after MI (2–7 days), mainly due to cardiac rupture. Consequently, differences between groups were reflected primarily in mortality rather than in cardiac function among surviving animals.

Previous work from our group and others has shown that 5-HT7R signaling regulates macrophage morphology, motility and cytokine secretion in vitro and in vivo [10]. Extending these observations, our data suggest that 5-HT7R activation modulates the inflammatory phenotype of cardiac macrophages during the acute phase after MI. Consistently, LP-211 treatment altered macrophage composition within the infarcted myocardium and was associated with reduced inflammatory marker expression, supporting the concept that 5-HT7R signaling contributes to fine-tuning of post-infarction inflammation.

To gain mechanistic insight, we performed transcriptomic profiling of cardiac macrophages isolated 3 days after MI. RNA-sequencing revealed extensive transcriptional reprogramming upon both genetic deletion and pharmacological activation of 5-HT7R, with a substantial overlap of differentially expressed genes in the two conditions. Pathway enrichment analysis highlighted inflammatory signaling, chemotaxis, angiogenesis and wound healing processes as key targets of 5-HT7R modulation. Notably, LP-211 treatment reduced expression of IL-6, a cytokine strongly implicated in adverse post-MI remodeling and poor outcome [42–44], suggesting a potential mechanism underlying the observed survival benefit.

Among the differentially regulated genes, we identified *S100A9* and *Got1* as particularly interesting candidates. Both genes have been previously linked to MI pathology but had not been connected to serotonergic signaling. [45, 46]. *Got1* plays a central role in cellular amino acid metabolism and has been associated with improved survival in MI patients [46]. In line with our observation of reduced *Got1* expression in PBMCs from MI patients early after infarction, Mouton et al. reported decreased *Got1* levels in the remote myocardium of C57BL/6J mice at 3 and 7 days after MI, whereas *Got1* expression was increased in macrophages within the infarct scar at later stages [47]. Moreover, high *Got1* levels in the blood plasma of patients with acute MI have been associated with improved survival, supporting the concept of *Got1* as a potential biomarker and therapeutic target [46].

In our study, pharmacological activation of 5-HT7R was associated with increased *Got1* expression in cardiac macrophages during the acute phase after MI, suggesting that serotonergic signaling may support the maintenance of *Got1* expression under inflammatory conditions. We therefore propose that 5-HT7R signaling may limit inflammation-induced metabolic reprogramming of immune cells, thereby contributing to the preservation of *Got1* levels and facilitating resolution of inflammation. Consistent with this notion, activation of 5-HT7R signaling in the acute phase after MI could attenuate inflammatory metabolic rewiring of macrophages and PBMCs [48–50], which has been shown to suppress *Got1* expression, and thereby potentially influence post-infarction outcome. At the mechanistic level, these effects may involve activation of Gαs-cAMP-PKA-CREB axis and ERK/mTOR pathways, which possibly attenuate NF-κB/HIF-1α–driven glycolytic polarization and maintain malate-aspartate shuttle activity [51–56]. As a result, *Got1* expression remains preserved, supporting cell metabolism and effective resolution of inflammation.

Interestingly, we observed a correlation of 5-HT7R expression with *S100A9* levels in PBMCs. We also show that *S100A9* expression is significantly upregulated in human PBMCs during the acute phase after MI. Consistent with these findings, we detected increased *S100A9* levels in macrophages isolated from the hearts of 5-HT7R-KO mice 3 days post-MI compared to WT mice. *S100A9*-expressing macrophages play a critical role in the early inflammatory response after MI by amplifying tissue damage, while also contributing to the transition toward a reparative phase through promotion of fibrosis [32]. Experimental administration of S100A9 induces neutrophil and macrophage infiltration, modulates macrophage polarization, enhances infarct wall thinning and reduces ejection fraction in mice [5, 24]. In line with these reports, we observed a significantly reduced ejection fraction in 5-HT7R-KO mice 14 days after MI compared to WT mice, which may be linked to elevated *S100A9* expression in cardiac macrophages during the acute phase 3 days post-MI.

Importantly, previous studies demonstrated that short-term inhibition of S100A9, either by pharmacological blockade or macrophage-targeted *S100A9* siRNA, restores key biological processes that are otherwise impaired after MI [4, 22]. In contrast, prolonged inhibition of *S100A9* negatively affects cardiac repair and counteracts the beneficial effects of short-term intervention, underscoring the importance of precise temporal control of *S100A9* activity during the dynamic inflammatory phases following MI [24].

Importantly, we were able to highlight a potential clinical relevance of targeting 5-HT7R signaling by demonstrating that isolated PBMCs from human patients’ blood in the acute phase (3±1 days) after MI showed regulated mRNA expression levels of *Got1* and *S100A9*, which positively correlated with 5-HT7R expression levels.

## Limitation of the study

Certain limitations should be acknowledged. 5-HT7R signaling is known to impact various physiological functions, including thermoregulation, circadian rhythm, learning, sleep, and more. Therefore, 5-HT7R agonists, such as LP-211, can possibly exert effects on recovery after MI through a plethora of processes, and might not be limited to the regulation of macrophages. Further, we did not uncover how LP-211 treatment positively regulates survival without improving cardiac function, and if effects would be consistent in female mice.

## Conclusions

In summary, we demonstrate that 5-HT7R signaling is transiently upregulated in inflammatory cells after MI and plays a critical role in shaping the post-infarction inflammatory response. Loss of 5-HT7R signaling aggravates cardiac dysfunction, whereas pharmacological activation improves survival after MI. These effects are associated with modulation of macrophage gene expression programs, including pathways linked to inflammation and metabolism. Our findings identify 5-HT7R as a novel and potentially druggable target to fine-tune immune responses and improve outcomes after MI.

## Electronic supplementary material

Below is the link to the electronic supplementary material.

Supplementary Material 1

Supplementary Material 2

Supplementary Material 3

## Data Availability

The datasets used and/or analysed during the current study are available from the corresponding author on reasonable request.

## Abbreviations

5-HT system: Serotonergic system
5-HT7R: Serotonin receptor 7
5-HT7R-KO: 5-HT7R-knockout
Adgre1: Adhesion G protein-coupled receptor E1
ACE: Angiotensin-converting enzyme
ANP: Atrial natriuretic peptide
bpm: Beats per minute
BW: Body weight
DEGs: Differentially expressed genes
Echo: Echocardiography
FAC: Fractional area change
FPKM: Fragments per kilobase of transcript per million mapped reads
FS: Fractional shortening
GO: Gene ontology
Got1: Glutamic-Oxaloacetic Transaminase 1
H&E: Hematoxylin and eosin
HBSS: Hanks balanced salt solution
HF: Heart failure
HPRT: Hypoxanthine-guanine phosphoribosyltransferase
HW: Heart weight
i.p.: Intraperitoneally
IB4: Isolectin B4
IHC: Immunohistochemistry
KEGG: Kyoto Encyclopedia of Genes and Genomes
LAD: Left anterior descending artery
LV: Left ventricular
LVEDD: LV end-diastolic diameter
LVESD: LV end-systolic diameter
LW: Lung weight
MACS: Magnetic-activated cell sorting
MDD: Major depressive disorders
MI: Myocardial infarction
ORA: Over-representation analysis
PBMCs: Peripheral blood mononuclear cells
PCI: Percutaneous coronary intervention
PSLAX: Parasternal long-axis
RNA-Seq: RNA-sequencing
RTqPCR: Quantitative realtime PCR
S100A9: S100 Calcium Binding Protein A9
WGA: Wheat germ agglutinin
WT: Wildtype
WT+LP-211: LP-211-treated WT mice

## Declarations

### Animal experiments

All animal studies were conducted in accordance with German animal protection law and with the European Communities Council Directive 86/609/EEC and 2010/63/EU for the protection of animals used for experimental purposes under consideration of the ARRIVE guidelines. All experiments were approved by the local institutional animal care and research advisory committee and permitted by local authority, the Niedersächsisches Landesamt für Verbraucherschutz und Lebensmittelsicherheit (application number 33.19–42502-04–18/2945, 33.942502-04–08-1575, 33.9–42502-04–06/1211, 33.9–42502-04–06/1112).
