# Supplementary material for: Serotonin 5-HT7 receptor signaling modulates inflammatory responses and survival after myocardial infarction
Source: J Transl Med. 2026 Jun 17;24:787. doi: 10.1186/s12967-026-08423-4 (PMC13281426; doi:10.1186/s12967-026-08423-4)
Supplement: Supplementary file 3 — Supplementary Material 3 [file 12967_2026_8423_MOESM3_ESM.docx]

**Supplementary Data**

**Serotonin 5-HT7 receptor signaling modulates inflammatory responses and survival after myocardial infarction**

Franziska E. Müller^1*^, Frauke S. Bahr^1*^, Sergej Erschow^2^, Martina Kasten^2^, Maren Heimerl^2^, Nils Benen^1^, Mira Jung^3^, Arne Schmidt^3^, Michaela Scherr^4^, Christine S. Falk^5^, Christian Bustamante^6^, Igor Ponomarev^7,8^, Johann Bauersachs^2^, Denise Hilfiker-Kleiner^2,9^, Thomas Thum^3^, Evgeni G. Ponimaskin^1#^ and Melanie Ricke-Hoch^2#^

* - authorship note: Franziska E. Müller and Frauke S. Bahr contributed equally as first authors

\# - authorship note: Evgeni G. Ponimaskin and Melanie Ricke-Hoch contributed equally as senior authors

**Corresponding author**:

Melanie Ricke-Hoch

Dept. of Cardiology and Angiology

Hannover Medical School

Carl-Neuberg Str. 1

30625 Hannover, Germany

Phone: +49-511-532-2531, Fax: +49-511-532-3263

E-mail: hoch.melanie@mh-hannover.de

**Supplementary figures**

**Supplementary Fig. S1** Validation of the MI model and expression of the 5-HT7R signaling partners. Relative mRNA expression levels of (A) atrial natriuretic factor (ANP), (B) cluster of differentiation 80 (CD80), and (C) CD206 normalized to HPRT (hypoxanthine-guanine phosphoribosyltransferase) in cardiac scar and remote regions 3, 7, and 14 days after sham or MI-surgery (3d Sham, 7d Sham, 7d MI: N=5; 3d MI, 14d MI: N=8; 14d Sham: N=11) . (D) Quantification of CD44, matrix metallopeptidase 9 (MMP9), GαS, Gα12, cell division control protein 42 homolog (Cdc42), ras homolog family member A (RhoA). Expression normalized to fragments per kilobase of transcript per million mapped reads (FPKM) in macrophages basal, 1 and 7d post-MI (N=3). Statistical analysis was performed using ordinary One-Way ANOVA with Šídák multiple comparison post hoc test (A-C) and tukey multiple comparison post hoc test (D).

**Supplementary Fig. S2** Altered chemokine profile in 5-HT7R-KO mice hearts 3 days after MI (A) Principal component (PC) analysis of secreted analytes in the apex of WT and 5-HT7R-KO mice 3 days post sham- or MI-surgery. (B) Fold changes of secreted chemokines and cytokines in WT and 5-HT7R-KO 3 days following sham-operation or MI-surgery relative to sham-operated WT animals (WT Sham, 5-HT7R-KO Sham: N=5; WT MI, 5-HT7R-KO MI: N=8). White crossed out boxes (X) indicate values under the detection limit of the standard curve of each analyte.

**Supplementary Fig. S3** LP-211 treatment modulates macrophage composition in cardiac tissue 14 days post-MI. Representative images highlighting 5-HT7R (green), CD68 (yellow), CD80 (red), and CD206 (turquoise) expression as well as nuclei (DAPI; blue) in the (A) border and (C) remote region in WT, 5-HT7R-KO and WT treated with LP-211 (WT+LP-211) (N=26) 14 days after surgery. Scale bars 50 μm. Quantification of sum intensity of 5-HT7R and macrophage marker expression in (B) the border and (D) remote area 14 days after surgery (WT: n=40 slices from 10 animals; 5-HT7R-KO: n= 28 slices from 5 animals; WT+LP-211: n=60 slices from 15 animals). Statistical analysis was performed using Kruskal-Wallis with Dunn’s multiple comparison test (B, D).

**Supplementary Fig. S4** LP-211 treatment increases angiogenesis in the border zone 14 days after MI. (A) Representative images of CD31^+^ endothelial cells (yellow), Nestin (red) and nuclei (DAPI; blue) in WT, 5-HT7R-KO and WT+LP-211 14 days post-MI. Scale bars 50 μm. (B) Quantification of mean intensity of CD31 and Nestin expression in all groups 14 days after surgery (n=20 slices from N=5 animals per group). Scale bars 50 μm. Statistical analysis was performed using Kruskal-Wallis with Dunn’s multiple comparison test (CD31) and ordinary One-Way ANOVA with tukey multiple comparison post hoc test (Nestin).

**Supplementary Fig. S5** Hypertrophic growth and collagen compositions are unaffected by LP-211 treatment post-MI. (A) Representative of WGA (green), Isolectin B4-fluorescein (IB4; red), and nuclei (blue) in the remote LV regions in WT, 5-HT7R-KO and WT+LP-211 animals 14 days after MI. Scale bars 50 µm. (B) Dot plots showing evaluation cardiomyocyte number and area, number of capillaries and capillary to cardiomyocyte ratio in all three groups (N=6). (C) Representative images of Sirius red stained scar region of WT, 5-HT7R-KO, and WT+LP-211 animals 14 days post-MI (N=5). Scale bars 100 μm. (D) Quanitifcation of fraction of collagen fibers 14 days after surgery (N=5 per group). Statistical difference was assessed using (B) ordinary One-Way ANOVA with Tukey’s multiple comparisons test and (D) using two-way ANOVA.

**Supplementary Fig. S6** Screening of DEGs and impact of 5-HT7R-KO on macrophage inflammatory profile 3 days post-MI. (A) Principal component (PC) analysis showing equal distribution of used samples. (B) Vulcano plots for differently expressed genes comparing WT to WT treated with LP-211 (left) and 5-HT7R-KO to WT. Red dots are up regulated genes and and blue dots indicate down regulated genes. (C) Heatmap indicates differently expressed genes between WT and 5-HT7R-KO. (D) Dotplot of Gene ontology (GO) and Kyoto Encyclopedia of Genes and Genomes (KEGG) enrichment analysis showing differenly regulated pathways in 5-HT7R-KO animals in comparison to WT post-MI, plotted against the gene ratio. The size of the dots represents the count of differentially expressed genes within the pathway and the coulor the p-value. (E) Gene enrichment analysis of differentially expressed genes in 5-HT7R-KO in comparison to WT 3 days after MI. Pathways are indicated in yellow. Upregulated genes are indicated in red, and downregulated genes are blue.

**Supplementary tables**

**Supplementary Tab. S1** Basal echocardiography in male 5-HT7R-KO mice. Echocardiographic analysis was performed in 14-16 weeks old male 5-HT7R-KO and corresponding WT mice. Data are depicted as mean ± SD or as median with interquartile range (IQR) depending on gaussian distribution. Statistical analysis was done using unpaired *t*-test (FAC, FS, LVEDD, LVESD, HR) and Mann Whitney test (LVEDA, LVESA), respectively.

| **Parameters** | **WT**  N=56 | **5-HT7R-KO**  N=48 |
| --- | --- | --- |
| FAC [%] | 48.2 ± 7.3 | 48.8 ± 7.4 |
| LVEDA [cm^2^] | 0.222 (0.200-0.251) | 0.217 (0.206-0.238) |
| LVESA [cm^2^] | 0.115 (0.102-0.130) | 0.109 (0.100-0.124) |
| HR [bpm] | 590 ± 32 | 591 ± 39 |
| FS [%] | 37.0 ± 5.5 | 37.7 ± 3.9 |
| LVEDD [mm] | 4.37 ± 0.36 | 4.37 ± 0.33 |
| LVESD [mm] | 2.75 ± 0.31 | 2.72 ± 0.24 |
| HR [bpm] | 592 ± 31 | 592 ± 40 |

FAC, fractional area change; LVEDA, left ventricular enddiastolic area; LVESA, left ventricular endsystolic area; HR, heart rate (bpm, beats per minute) determined in parasternal long-axis (PSLAX) view recorded in B-mode. FS, fractional shortening; LVEDD, left ventricular enddiastolic diameter; LVESD, left ventricular endsystolic diameter measured in short-axis view recorded in B-mode; HW, heart weight; BW, body weight; LW, lung weight.

**Supplementary Tab. S2** Cardiac function and dimensions in male 5-HT7R-KO and WT mice 3 days after MI. Echocardiographic analysis was performed in 14-16 weeks old male 5-HT7R-KO and corresponding WT mice. Data are depicted as mean ± SD or as median with interquartile range (IQR) depending on gaussian distribution. Statistical analysis was done using unpaired *t*-test (FAC, LVEDA, LVESA, HR, HW, HW/BW) and Mann Whitney test (BW, LW, LW/BW), respectively.

| **Parameters** | **WT sham**  N=5 | **5-HT7R-KO sham**  N=5 | **WT MI**  N=9 | **5-HT7R-KO MI**  N=9 |
| --- | --- | --- | --- | --- |
| FAC [%] | 45.0 ± 9.3 | 47.9 ± 6.5 | 18.4 ± 11.8 | 13.3 ± 6.1 |
| LVEDA [cm^2^] | 0.228 ± 0.010 | 0.235 ± 0.028 | 0.310 ± 0.021 | 0.323 ± 0.032 |
| LVESA [cm^2^] | 0.126 ± 0.024 | 0.123 ± 0.025 | 0.254 ± 0.045 | 0.281 ± 0.039 |
| HR [bpm] | 619 ± 33 | 598 ± 46 | 600 ± 25 | 588 ± 41 |
| HW [mg] | 121.7 ± 14.4 | 134.5 ±12.5 | 155.0 ± 16.4 | 152.2 ± 10.8 |
| BW [g] | 26.7 (26.1-27.5) | 27.4 (26.7-28.4) | 25.0 (24.7-26.1) | 24.7 (22.9-26.5) |
| HW/BW [mg/g] | 4.54 ± 0.4 | 4.89 ± 0.4 | 6.07 ± 0.7 | 6.16 ± 0.5 |
| LW wet [mg] | 139.4  (126.3-145.0) | 128.2  (122.2-148.0) | 145.2  (132.2-169.7) | 149.6  (129.2-156.3) |
| LW dry [mg] | 33.6 (30.7-34.4) | 31.7 (30.3-35.8) | 34.4 (32.7-39.8) | 34.4 (31.4-35.6) |
| LW wet/BW [mg/g] | 5.14 (4.73-5.45) | 4.82 (4.41-53.6) | 5.42 (5.10-6.79) | 5.75 (5.50-6.55) |

FAC, fractional area change; LVEDA, left ventricular enddiastolic area; LVESA, left ventricular endsystolic area; HR, heart rate (bpm, beats per minute) determined in parasternal long-axis (PSLAX) view recorded in B-mode; HW, heart weight; BW, body weight; LW, lung weight.
